# Automated Data Merging, Analysis and Structure Solution in RAPD2

**DOI:** 10.1063/4.0001036

**Published:** 2025-10-27

**Authors:** Kay Perry, Frank V Murphy, David Neau, Jonathan Schuermann

**Affiliations:** 1 Cornell University, Ithaca, NY, USA; 2 Northeastern Collaborative Access Team, Lemont, IL, USA; 3 Northeastern Collaborative Access Team, Lemont, NY, USA

## Abstract

In the current regime of fast data acquisition and vast computing power, automated data processing coupled to data analysis and structure solution should be the norm. RAPD2 (Rapid Automated Processing of X-ray Data 2) at the Northeastern Collaborative Access Team (NE-CAT) combines Python 3.0 on the backend and the AngularJS framework on the frontend to create an updated user interface that works on any current web browser to allow users to leverage the computing resources at NE-CAT to view processed data, data analysis and perform structure solution. Coming off the APS-U, NE-CAT has modernized our heavily used RAPD away from Python2.7 and PHP; but the basic concept remains the same. In the data analysis and structure solution pipelines, RAPD2 leverages the power of python and CCTBX (the Computational Crystallography Toolbox) to automatically launch crystallographic programs, scrapes the results from program output and presents the results to the users on a web-browser, eliminating the need for installation of programs.

After automated data integration, RAPD2 offers data analysis through a combination of programs from CCP4 and Phenix. The space group of the dataset is verified with pointless, checked for pseudo-translation, twinning and non-crystallographic symmetry. Datasets can be assessed for isomorphism through either similarity in Bragg reflection intensities or unit cell variation and then merged to increase anomalous signal or to complete partial datasets. Molecular replacement using CCP4 and Phenix is available using a known structure or AlphaFold model. SAD phasing with autobuilding leverages Shelx and Phenix. Ligand detection through peak search of a difference map is also available. With designation of projects, in the future, data merging and structure solution can also be automated, providing users with a structure that only requires model building and refinement at the end of a synchrotron visit.

